# Using the incidence and impact of behavioural conditions in guide dogs to investigate patterns in undesirable behaviour in dogs

**DOI:** 10.1038/srep23860

**Published:** 2016-04-14

**Authors:** Geoffrey Caron-Lormier, Naomi D. Harvey, Gary C. W. England, Lucy Asher

**Affiliations:** 1 School of Veterinary Medicine and Science, University of Nottingham, Sutton Bonington Campus, Leicestershire, LE12 5RD, UK; 2 Centre for Behaviour and Evolution, Henry Wellcome Building, Newcastle University, Newcastle, NE2 4HH, UK

## Abstract

The domestic dog is one of our most popular companions and longest relationships, occupying different roles, from pet to working guide dog for the blind. As dogs age different behavioural issues occur and in some cases dogs may be relinquished or removed from their working service. Here we analyse a dataset on working guide dogs that were removed from their service between 1994 and 2013. We use the withdrawal reasons as a proxy for the manifestation of undesirable behaviour. More than 7,500 dogs were in the dataset used, 83% of which were retired (due to old age) and 17% were withdrawn for behavioural issues. We found that the main reasons for behaviour withdrawal were environmental anxiety, training, and fear/aggression. Breed and sex had an effect on the odds of dogs being withdrawn under the different reasons. The age at withdrawal for the different withdrawal reasons suggested that dogs were more likely to develop fear/aggression related issues early on, whilst issues related to training could develop at almost any age. We found no evidence for heterosis effecting behaviour. We believe that this work is relevant to the pet dog population and had implications for understanding ageing and genetic influences on behaviour.

The domestic dog is one of our most popular companion animals inhabiting 24–31% of households in the UK^1^, 36% in Australia, and 40% in the USA^2^. In addition to companionship, the domestic dog occupies a multitude of roles including guiding the blind or visually impaired^3^, as police or military dogs^4^,^5^, for the detection of hazardous substances such as drugs or explosives^6^, as assistance dogs for the disabled^7^, and even as epilepsy detection dogs^8^. Significant effects of sex and breed have been found on the incidence of health issues, which lead to early withdrawal of qualified guide dogs^9^, however little is known about the impact of age, sex and breed on the manifestation of undesirable behaviour in guide dogs.

In a cross-sectional study of lifespan development of attentiveness in dogs, differences in attentional performance were documented, with both young and particularly old dogs demonstrating deficiencies in attentional control^10^. These results suggest that older dogs could be more likely to develop undesirable behaviours relating to increased distractibility, which could in turn contribute to a breakdown in trainability and obedience. A recent cross-sectional study of Swedish dog breeds showed small, but statistically significant differences in mean scores on behavioural traits scored by the C-BARQ (a validated dog behaviour questionnaire), between breeds and breed groups, with trait dependent age and sex differences, in addition to breed by age and breed by sex interactions on certain trait scores^11^.

Studies of puppies and young adult dogs, below two years of age, show that different breeds reach behavioural maturity at different rates^12^. If such differences continue into adulthood, breed by age interactions may occur in the manifestation of undesirable behaviour in adult and ageing dogs. Evidence of behavioural sexual dimorphism in the dog could further impact differences in the development of undesirable behavioural. Adult female dogs (more than one year of age) are often less aggressive, more fearful (less bold or confident), more excitable, more distractible and more trainable than males^11^,^13^,^14^,^15^,^16^,^17^.

Evaluation of dog behaviour has been of great interest to working dog organisations, and prediction of behavioural suitability to the working role has been the focus of much study; with progression from training to a working role as the outcome of interest^3^,^4^,^5^,^6^,^18^. The behavioural reasons for withdrawal from training prior to qualification are well defined, with many being attributed to distractions^19^, or fearfulness^13^,^20^. However, few studies have continued to study working dog behaviour past the point of completion of training, which is also known as qualification. Following qualification, the reasons for break down of a partnership between a guide dog and its owner can be complex, often dependent upon the owner’s expectations, the quality of the match and of the relationship between the dog and owner, and the dogs working performance^21^. Little is known about the impact of behavioural issues with the dog in these breakdowns.

In this study, we consider the age at which undesirable behaviour manifests, and the associated reduction in working life, in a population of working guide dogs. We consider undesirable behaviour to be the appropriate term in this context; whilst behaviour may be species-appropriate (e.g., coprophagia), it is undesirable to their owner or caregiver. Early retirement of a working dog due to a behavioural issue is considered as a proxy measure of an emergent undesirable behaviour. Whilst the behavioural issue may have begun at an earlier stage in life, the point at which the dog was withdrawn (and not retrained) is a marker for an undesirable behavioural that has become unmanageable to the extent that the dog is considered to no longer adequately perform its working role. The impact of four main external factors on the manifestation of behavioural issues are considered; age, sex, breed and heterosis (based on pure and crossbreed generation).

Our aims were to investigate the main behavioural reasons for withdrawal from work of qualified guide dogs, quantify the impact each of these reasons upon the length of working life as compared to dogs that retire due to old age, and identify potential links between withdrawal from work for behavioural reasons and the dog’s age, breed and sex. To do so, we used a large retrospective dataset of information on working guide dogs, Guide Dogs (UK), that had either retired due to old age or were withdrawn from work before reaching retirement age due to undesirable behaviour.

## Material and Methods

### Guide Dogs data

Guide Dogs (UK) is the current working name of the Guide Dogs for the Blind Association. It started in 1931 and is now the “world’s largest breeder and trainer of working dogs”^22^. Guide Dogs can breed around 1,300 puppies every year, most of which will go through training and then be paired with a visually impaired person when they are about two years of age.

There are five stages in the training of a guide dog: (1) breeding, the majority of dogs trained by Guide Dogs are bred by the organisation and undergo extensive, standardised socialisation at the National Breeding Centre between the ages of 6–8 weeks; (2) puppy walking, during which the dogs live with a volunteer carer from the age of 8 weeks until approximately 12–14 months; (3) early training, where dogs enter kennels (at approximately 14 months of age) and undergo basic guide dog training (most dogs are neutered by this stage); (4) advanced training, where dogs are trained in advanced guiding skills (at 18 months of age); (5) partnership training, the final stage of training where the dog and its guide dog owner (GDO) are trained to work together as a unit (at appoximately 20 months of age). See more details at this link http://www.guidedogs.org.uk/microsites/sponsor-a-puppy/about-sponsor-a-puppy/puppy-training.

Once paired with a GDO, dogs may reach a retirement due to old age, this is the case for the majority of qualified dogs (~70%) which are retired after 8.5 years of working as a guide dog at approximately 10.5 years of age^9^. A small number of dogs may be retired from work because their GDO can no longer keep them, at which point they may be re-matched with another GDO. If re-matching is not possible (for example if the dog had been with its original GDO for a long period of time) it may be retired and re-homed. The remaining dogs that fail to reach retirement age are withdrawn due to health (~14%) or (~16%) behavioural issues. Guide Dogs have measures in place to minimise the impact of withdrawals on guide dog owners.

Dogs were included in this study if they had, first qualified, and second been matched by Guide Dogs (UK) with a person who is blind or partially sighted. All dogs were withdrawn for behavioural issues or were retired due to old age between 1 January 1994 and 31 December 2013 (a 20-year period). Most guide dogs in this sample were bred by Guide Dogs’ breeding program, but a minority would be sourced from breeders of relevant breeds. All dogs were neutered.

The data were collated and maintained by Guide Dogs’ staff. The identification of a behavioural problem was performed by a trained, and certified, member of Guide Dogs’ staff (known as a Guide Dog Mobility Instructor trained through an internal qualification of international standard) based on feedback, and consultation with the Guide Dog Owner. The decision to withdraw the dog is made by a number of Guide Dogs staff in consultation with the Guide Dog Owner with consideration to human safety, dog welfare, and the dog’s ability to perform its working role with their current GDO. Dogs with undesirable behaviour that was judged as correctable (e.g., excessive barking, recall response) using training would not be withdrawn. Dogs judged to be able to perform the working role with a different GDO (e.g. one with different needs, visual impairment or home environment) would be retrained and not withdrawn. The study was approved by Guide Dogs and the University of Nottingham’s ethical review process, and the methods were carried out in accordance with the approved guidelines.

### Classification

In this study we were interested in the behavioural reasons for withdrawal after dogs had been paired with a blind or partially sighted owner. Dogs were considered to be retired if the reason provided for withdrawal of the dog was ‘old-age’, and there was no indication of behavioural (or health) deterioration which effected their functioning as a working guide dog. For all dogs their (total) working life was recorded as a number of days; that is the time between first qualification and end of service.

The dataset contained 7,770 dogs that either were withdrawn for behavioural reasons or reached old age (i.e., retirement). Dogs with parent stock from the same breed were labelled as “PureBreed” to distinguish them from cross breed dogs which had parent stock from more than one breed (indicated by a * when parent breeds are not written in full). We considered the eight most common breeds (95% of the population) for analysis and grouped the rest (5%) into an ‘Other’ category. The numbers, and abbreviations, are as follows: Labrador (L, 2877), Golden Retriever x Labrador (GRxL, 2167), Golden Retriever (GR, 848), Labrador x Golden Retriever (LxGR, 741), Other breeds (Other, 374), German Shepherd Dogs (GSD, 286), F2 Labrador x Golden Retriever (LxGR*, 268), F2 Labrador cross (LxL*, 116), F2 Golden Retriever cross (GRxGR*, 93). We grouped the breeds GRxGR* and LxL* into Other to avoid singularities in the statistical analysis. GRxL is where the Golden is the dam and the Labrador the sire. The sex of a dog was either male or female; neutered status was not considered since all dogs were neutered.

The behavioural withdrawal reasons were categorised into 10 groups. These groups were based on a factor analysis conducted by a member of our group (Asher, unpublished data). We removed the grouping “body sensitivity” from the analysis due to the limited number of cases to analyse. Additionally, we removed seven dogs due to inconsistencies in data recording. The behaviour withdrawal reasons were the outcomes (or variables) of interest for this analysis and the numbers of dogs in each withdrawal group are detailed in Table 1. We also list all the specific reasons for withdrawal, and their associated groups, in the appendix.

### Data analysis

All statistical and numerical analyses were conducted in R 3.2.x^23^. We considered two outcome variables: (1) the incidence of withdrawals due to undesirable behaviours in the 10 different behavioural groups; (2) the total length of working life. We considered the effects on these outcome variables of three predictors of interest: PureBreed, Breed and Sex. We define the incidence as the number of cases (i.e., the number of dogs) withdrawn for the reasons in each of the behavioural groups within the study period^24^.

The Pearson’s *χ*^2^ Test for Count Data was used to test for independence (in *R, chisq.test*()) between each of the different factors of interest (PureBreed, Breed and Sex) and to consider differences in incidence of the 10 behavioural groups. These univariate tests were used to determine whether to include the factors in the subsequent logistic regressions.

The logistic regression, generalised linear model, was used to test for the likelihood of dogs to be withdrawn under each of the 10 behavioural groups, in turn. We used the *glm*() function with a *binomial* family. We checked for the impact of the predictors which were significant in the univariate analysis. We also tested for the interactions between Breed and Sex. The logistic regressions were run as follows: the retired group (Old) was tested against all the other groups combined (i.e., withdrawn) thereby testing the likelihood of dogs reaching retirement, and how breed, or sex, may influence this. Then, each withdrawal group (e.g., Fear/Aggression) was tested in turn against the reference “Retired” thereby testing the likelihood of dogs being withdrawn under that particular behavioural group compared to the reference, and how Breed, or sex may influence this.

We used a linear model, using generalised least squares, to check for the difference in total working life between the behavioural groups and the influence of the two predictors (Breed and sex). The working life was measured in days as the time between start and end of work. The references for the predictors were retired (for the withdrawal groups), Labrador (for the breeds) and bitch (or female, for the sex). Throughout the paper, we limited the results presentation to the significant results. When factor levels presented no, or few, significant results, they were omitted from the tables or figures but were cited in the text. However, all factor levels were kept in the analysis unless stated otherwise. We used a generalised least squares method because the standard linear (with least squares method) and the generalised linear models were not suitable due to the heterogeneity of the variance. We checked the model fits by visually inspecting the distribution, and the homogeneity of variance, of the residuals. All fits were considered acceptable as described and recommended in Zuur *et al*.^25^.

## Results

### Summary statistics

The dataset contained 7,770 working guide dogs; 6,465 dogs reached the retirement age, which represents just above 83% of the population. The remaining 17% (1,305 dogs) were withdrawn for behavioural reasons and assigned to a behavioural group. The data contained 3,880 female and 3,890 male dogs. The pure breed factor was not significant in any analyses and will not be mentioned further.

### Incidence of behavioural group withdrawals

**Figure 1:**
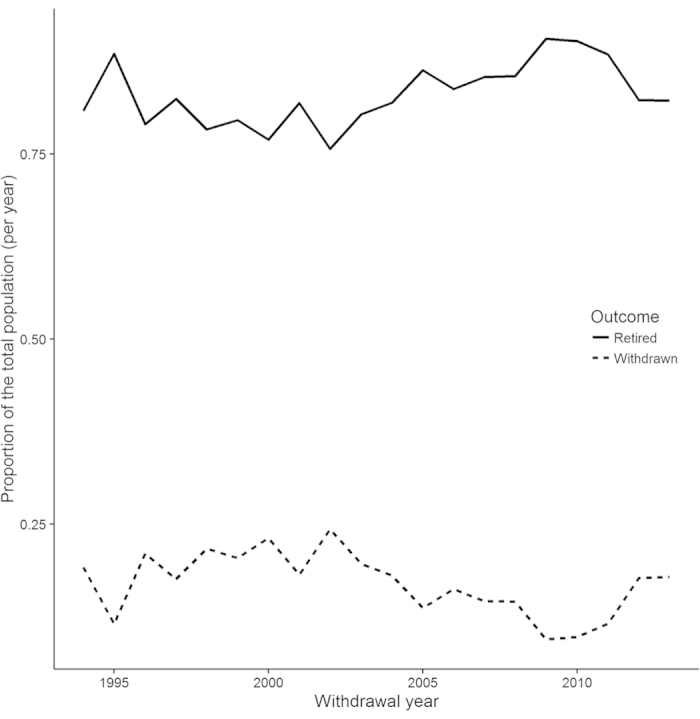
Proportions of dogs withdrawn and retired relative to the total population for each year in the data set.

**Figure 2:**
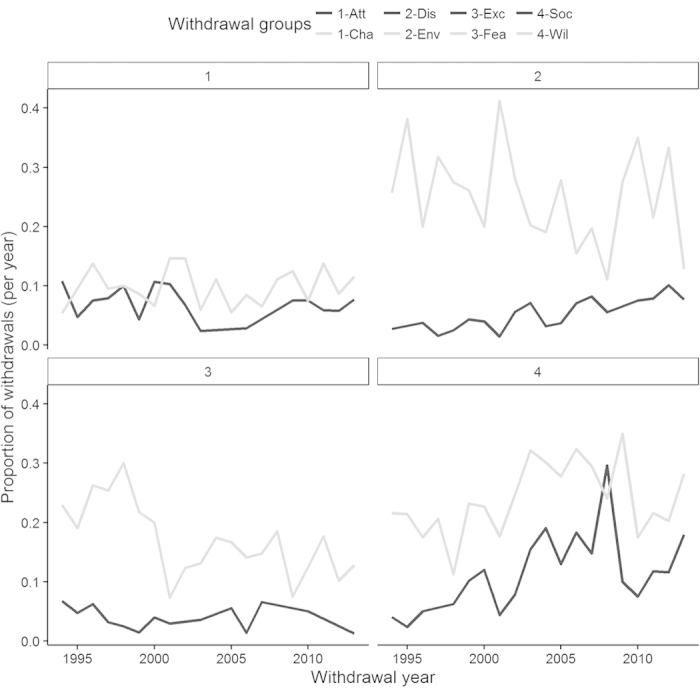
Proportion of dogs withdrawn in each withdrawal group over the 20-year time period of the study. The four different panes displayed have no biological significance.

Figure 1 illustrates the proprotions of dogs either withdrawn for behaviour issues or retired (for old age) over the years of the dataset. We find that the proportions seem stable over time with random fluctuations. Figure 2 illustrates the change over time of the proportions of the different withdrawal groups (proportions of all withdrawal, not including the retired dogs). We find that most proportions are stable, for instance Attentivness and Chasing. Whilst Distraction seems to be proportionally increasing, Environmental Anxiety seems to be descreasing.

The three main groups for which dogs were withdrawn from work were Environmental Anxiety (321 dogs), Willingness (311 dogs) and Fear/Aggression (226 dogs, Table 1). The behavioural group Distraction showed no significant results for any of the predictors from the *χ*^2^-test (Supplementary Table 1). Conversely, the Fear/Aggression group showed significant results for all predictors. The other behavioural groups returned a mix of significant results which suggested they might be important in the following analysis.

Table 2 lists most of the odd ratios from the generalised linear model between each of the withdrawal groups and the retired group. The predictors with few significant results are presented in the text below. Golden Retriever x Labrador had reduced odds of being withdrawn under the Excitability group (OR = 0.31, CI = [0.09:0.85], p < 0.05). Golden Retrievers showed increased odds of being withdrawn under the Environmental Anxiety group (OR = 1.49 CI = [1.03:2.12], p < 0.05) and decreased odds of being withdrawn under the Social Behaviour group (OR = 0.46, CI = [0.2:0.92], p < 0.05). We also note that German Shepherd dogs had reduced odds of reaching retirement age, thereby increasing their odds of being withdrawn under various behavioural groups, particularly the Fear/Aggression group (OR = 7.02, CI = [4.46:10.91], p < 0.001). The breed category “Other” showed increased odds for the withdrawal group Fear/Aggression (OR = 2.01, CI = [1.22:3.22], p < 0.01). We find that male dogs had increased odds of being withdrawn under the Chasing (OR = 1.69, CI = [1.18:2.43], p < 0.01) and Fear/Aggression (OR = 1.50, CI = [1.15:1.97], p < 0.01) groups.

### Impact on total working life

**Figure 3:**
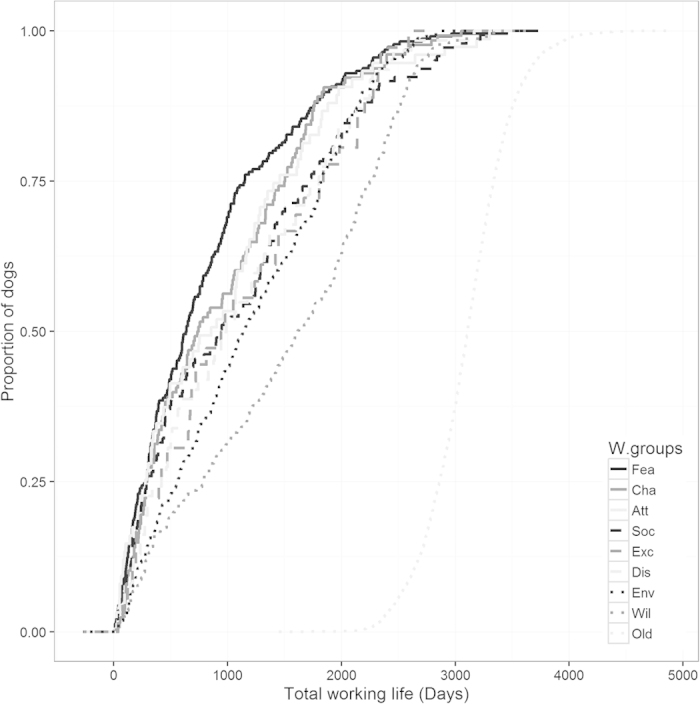
Proportion of dogs removed in their respective withdrawal groups of their working life in days.

**Figure 4:**
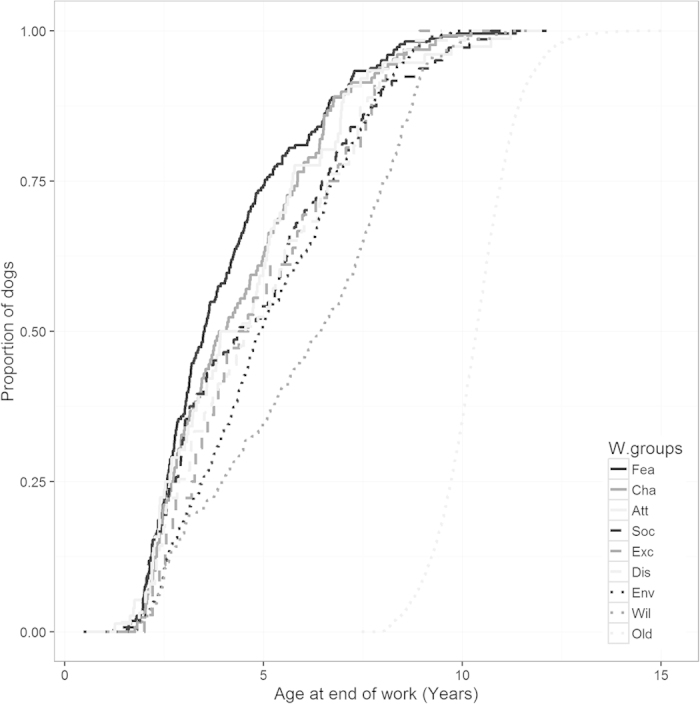
Proportion of dogs removed in their respective withdrawal groups as dogs age.

The overall mean working life for the retired category was 3,097 ± 6.38 days (Fig. 3). All other withdrawal groups were significantly different (*p* < 0.001) with loss of working life ranging from 2,286 days (s.e. of 47) to 1,580 days (s.e. of 49). We found that sex had no significant effect on the mean working life. Similarly, the level of cross between breeds (pure, F1, F2 and F3) showed no impact on mean working life. Figure 4 illustrates the same point but using the age at withdrawal instead of the total working life.

When taking into account the dog breeds, we find that the overall mean working life for a Labrador in the retired category was 3,108 days (s.e. 7). The only two breeds showing a difference were German Shepherds with a loss of 172 days (or about minus six months) (s.e. 26, *P* < 0.001) and Golden retrievers, which had a working life 36 days shorter on average (s.e. 15, *P* = 0.019). More details can be found in Supplementary Table 2.

### Age and behavioural issues

Age at end of work and total working life are highly correlated (Spearman’s rank correlation rho = 0.976, P < 0.001). Table 3 lists the withdrawal groups and their associated median of total working life (in years and days) and age at withdrawal. These numbers suggest that 50% of the dogs, in the Fear/Aggression group, will be withdrawn by the time they worked for under two years, or by the time they are 3.5 years of age. Conversely, the withdrawal group Willingness has a much higher associated median, with 4.4 years in working life and 6.4 years of age. Withdrawal groups Attentiveness, Social Behaviour and Excitability, have similar characteristics with a median age of ~4.5 years. More details can be found in Table 3 and Fig. 4.

## Discussion

The aims of this study were to investigate the main behavioural reasons for withdrawal of qualified guide dogs, to quantify the impact each of these reasons upon the length of working life, and to identify potential links between withdrawal from work for behavioural reasons and the dog’s age, breed and sex. To our knowlegde this is the first study of this kind. There were three main reasons for withdrawal for behaviour: Environmental Anxiety, Willingness and Fear/Aggression; the impact of behavioural withdrawals on length of working life varied according to the withdrawal group, ranging from a mean loss of 2,286 days to 1,580 days (compared to a mean working life for the retired group of 3,097 days). Interactions between withdrawal reason group, length of working life and the dogs’ sex and breed were in evidence and will be discussed in turn.

Whilst both sexes had equally long working lives, males were 50% more likely to be withdrawn for Fear/Aggression and Chasing than females. This supports previous findings that male dogs exhibit higher mean aggression levels than females^11^,^15^, although it is difficult to interpret the difference regarding withdrawals for chasing, as the aetiology behind chase proneness is unknown. Dogs’ motivations to chase could be based in play, excitement or potentially as part of an offensive or defensive aggressive response, or predatory behaviour.

No breeds were more likely to reach retirement than Labradors. However, German shepherd dogs (GSDs) and the Labrador x Golden retriever* F2 cross were 57% and 40% less likely to reach retirement than the Labrador, suggesting that these breeds display more undesirable behaviour to GDOs. GSDs were seven times more likely to be withdrawn for Fear/Aggression than Labradors. For male dogs this effect was additive, with male GSDs being up to 7.5 times more likely to be withdrawn for Fear/Aggression. However, GSDs were 70% less likely to be withdrawn for problems related to willingness and confidence. For example, Labradors were shown to be the least fearful of dogs assessed and German Shepherds the most^26^. Female puppies were more “independent” and “active” than male puppies, although behaviour was not consistent or predictive of future success^27^. Adult Labradors scored higher for “nerve stability”, had less “reaction to gunfire” and were more “cooperative” than German Shepherds^14^. The heritability value of these tests was 0.24, which was considered to be high^28^. Based on these results Labradors were suggested to be more suited to being a guide dog than German Shepherds.

Breakdowns in willingness related behaviours were more likely to occur later in life than behaviour problems related to excitability, chasing, attentiveness and social behaviour, which were more likely to occur earlier. Fear or aggression issues occurred in younger dogs than other withdrawal reasons. This group had the lowest median withdrawal days (635). This means that 50% of the dogs in that category had been withdrawn within two years after qualification. Dogs withdrawn for Chasing followed closely at 739 days. Conversely, those withdrawn for Willingness had a median of 1,617 days which indicates that issues under that category can occur 4–5 years after qualification (6.4 years old). It seems that willingness related issues can occur at any age with a steady slope shown for withdrawals in this category. Similarly, withdrawals for issues related to environmental anxiety can occur at any age.

For other undesirable behaviour, 2.5 years post-qualification, or approximately 4.5 years of age, seems to be the milestone beyond which behavioural issues are less likely to occur. This would suggest that the older the dogs get the more stable their behaviour becomes. However, we have no information on dogs that have been retired therefore we do not know if behavioural issues arise when dogs become geriatric. Similarly, we do not know whether withdrawals were caused by a gradual degradation of the partnership or by one significant event leading to the eventual withdrawal, as can sometimes be the case^29^.

Relinquishments of dogs to shelters in the US showed a significant trend to increase between 9 months and 6 years of age, with the most common age at relinquishment being between 1–2 years of age^30^ and the risk of relinquishment has been shown to decrease with increasing age^31^. However, 37% of dogs relinquished to these shelters were aged over 3 years of age^32^. The results of the current study provide evidence for age-associated risks of developing behavioural problems serious enough to stop a guide dog from working. Moreover, they allude to there being different trajectories for developing different types of behavioural issues. This is in line with previous applied behaviour findings^33^. Further research into the associations between ageing and the development of behavioural problems in dogs could be valuable; further understanding of these factors would allow for intervention or coping strategies to be implemented, potentially reducing the occurrence of shelter relinquishments for pet dogs, or withdrawal from work for working dogs.

Crossbred dogs often exhibit health benefits over their purebred counterparts^9^,^34^. Such benefits are evidence of genetic heterosis conferring an outbreeding advantage (hybrid vigour). In domestic dogs, closed gene pools within breeds have meant that dogs are more similar within a breed and therefore mixing with other breeds should increase genetic heterozygousity. Whilst evidence of hybrid vigour in regards to health (or production in farm animals) is well documented^35^,^36^,^37^,^38^, there is a current lack of published literature investigating potential differences in behaviour between pure and cross breeds. Within Guide Dogs, the breed with the best qualification rate is the first generation cross (F1) of the Golden Retriever and the Labrador^3^. However, the reason(s) why these crosses appear to be more suitable to guiding are, as yet, unknown.

Dog populations from Guide Dogs may be most amenable to the study of outbreeding advantage because of the extensive pedigree, health and behaviour information kept. In a previous paper, a population of qualified guide dogs was shown to exhibit signs of health-related outbreeding advantage, with crossbred dogs being more likely to have long healthy working lives than purebred dogs^9^. However, the current study revealed no association between behavioural withdrawals and the generation level of the dogs, from pure breed to second-generation crosses. This could suggest that behavioural hybrid vigour does not exist. However, dogs with severe behavioural issues were identified before qualification.

Heteroris in relation to health traits can be demonstrated because health problems tend to emerge as individuals age, whilst behavioural phenotypes are identifiable in young dogs or puppies^4^,^5^,^6^. Therefore, it is recommended that further investigation into potential behavioural differences between crossbreeds and their purebred parents be conducted using behavioural data collected on the entire population of dogs bred by Guide Dogs, or other organisations that breed their own dogs. Such an investigation could be highly relevant to the pet dog community, as well as working dogs, due to the current appetite for crossbreeds as pets amongst the general public.

Since the undesirable behaviour displayed by dogs in this population can also be shown in the general pet dog population it is assumed that these results might be applicable to some pet dog owners^39^,^40^,^41^. Indeed, the population evaluated here had already undergone a process of strict behavioural selection against dogs with traits unsuitable to guiding which were apparent during training, as is general practice for guide dogs^3^,^18^,^42^. This would suggest that the general dog population, which did not undergo such a process, could show more pronounced undesirable behaviour more often. Although the results are perhaps not applicable to the entire pet dog population, we believe that the overall patterns should be relevant.

## Conclusion

We believe that this work on working guide dogs could be relevant to the pet owner population and could have implications for understanding the effects of ageing on behaviour. The undesirable behaviours shown in the working guide dog population, such as anxiety and Fear/Aggression, are also seen in the pet population. Fear and aggression issues are a common reason for relinquishing younger dogs even if a pet owner might manage stronger intensities of these behaviours than a guide dog owner. Whilst stable behaviour traits have been identified in dogs, this data suggests that some behavioural traits in dogs changes with age.

## Additional Information

**How to cite this article**: Caron-Lormier, G. *et al*. Using the incidence and impact of behavioural conditions in guide dogs to investigate patterns in undesirable behaviour in dogs. *Sci. Rep.* **6**, 23860; doi: 10.1038/srep23860 (2016).

## Supplementary Material

Supplementary Information

## Figures and Tables

**Table 1:** Number of dogs in each combination of breed and behavioural withdrawal group.

W. Group	L	Other	GSD	GR	GRxL	LxGR	LxGR*
Retired	2427	487	200	712	1820	614	205
Attentiveness	30	7	7	5	17	5	5
Chasing	44	10	11	9	33	16	5
Distraction	19	8	2	8	20	3	3
Environmental Anxiety	103	19	18	45	84	36	16
Excitability	17	4	4	3	4	2	2
Fear/Aggression	60	24	34	18	60	17	13
Social Behaviour	59	6	7	8	44	12	8
Willingness/Confidence	118	18	3	40	85	36	11

**Table 2:** Odd ratios (and confidence intervals) from the generalised linear models of dogs ending service for the different withdrawal groups, and changes in odds based on dogs’ breed and sex.

W. Group	GSD	LxGR*	Reference
Retired	0.43*** [0.33:0.57]	0.6*** [0.45:0.82]	5.67*** [5.04:6.39]
Attentiveness	2.88* [1.15:6.28]	NS	0.01*** [0.01:0.02]
Chasing	3.13*** [1.51:5.95]	NS	0.01*** [0.01:0.02]
Distraction	NS	NS	0.01*** [0:0.01]
Environmental Anxiety	2.13** [1.23:3.5]	1.84* [1.03:3.09]	0.04*** [0.03:0.05]
Excitability	NS	NS	0.01*** [0:0.01]
Fear/Aggression	7.03*** [4.46:10.92]	2.59** [1.34:4.64]	0.02*** [0.01:0.03]
Social Behaviour	NS	NS	0.03*** [0.02:0.03]
Willingness/Confidence	0.3* [0.07:0.82]	NS	0.05*** [0.04:0.07]

P values are categorised as follows: ***p < 0.001, **p < 0.01, *p < 0.05. NS means Not Significant.

**Table 3:** Median total working life in days and in years, and age at withdrawal, of the dogs in the different withdrawal groups.

W. Group	Days	Years	Age
Fear/Aggression	635	1.74	3.50
Chasing	739	2.02	3.98
Attentiveness	855	2.34	4.46
Social Behaviour	945	2.59	4.39
Excitability	960	2.63	4.50
Distraction	986	2.70	4.71
Environmental Anxiety	1152	3.16	4.96
Willingness/Confidence	1617	4.43	6.44
Retired	3107	8.51	10.35
