# Supplementary material for: Using the incidence and impact of behavioural conditions in guide dogs to investigate patterns in undesirable behaviour in dogs
Source: Sci Rep. 2016 Apr 14;6:23860. doi: 10.1038/srep23860 (PMC4831008; doi:10.1038/srep23860)
Supplement: Supplementary Information [file srep23860-s1.pdf]

Using the incidence and impact of behavioural conditions in guide dogs to investigate undesirable behaviours in working dogs

Geoffrey Caron-Lormier[1], Naomi D. Harvey[1], Gary C.W. England[1], and Lucy Asher[2]

[1]School of Veterinary Medicine and Science, University of Nottingham, Sutton Bonington Campus, Leicestershire, LE12 5RD, UK
[2]Centre for Behaviour and Evolution, Henry Wellcome Building, Newcastle University, Newcastle, NE2 4HH, UK.

Table 1: Results of the χ^2-test for each of the variable PureBreed, Breed, Dog Sex, and Outside Bred. The variable PureBreed, the temperamental groups Body Sensitivity and Distraction were removed from the table as they returned no significant results. The values in bracket are the χ^2 and the degrees of freedom. Non-significant results ($P > 0.05$) are shown as NS.

Withdrawal Group	Sex	Breed
Attentiveness	NS (0.72,1)	0.021 (14.87,6)
Chasing	0.009 (6.78,1)	0.016 (15.68,6)
Environmental Anxiety	NS (0.1,1)	0.011 (16.46,6)
Excitability	NS (0.02,1)	0.03 (14,6)
Fear/Aggression	0.008 (6.95,1)	<0.001 (106.43,6)
Retired	NS (1.98,1)	<0.001 (48.85,6)
Social Behaviour	NS (0.45,1)	NS (10.81,6)
Willingness/Confidence	NS (2.09,1)	NS (8.16,6)

Table 2: Coefficients, and associated statistics, for the best least-squares regression of the working life showing the reduction in working life associated with each withdrawal and breed groups

Factor	Estimate	S.E.	t.value	p.value
Reference: Retired Labs	3108	7.343	423.3	<0.001
Behaviour:Attentiveness	-2124	91.82	-23.13	<0.001
Behaviour:Chasing	-2153	64.76	-33.24	<0.001
Behaviour:Distraction	-1993	101.4	-19.66	<0.001
Behaviour:Environmental Anxiety	-1891	42.08	-44.94	<0.001
Behaviour:Excitability	-1973	132.2	-14.93	<0.001
Behaviour:Fear/Aggression	-2264	47.55	-47.61	<0.001
Behaviour:Social Behaviour	-2010	72.91	-27.56	<0.001
Behaviour:Willingness/Confidence	-1584	49.26	-32.15	<0.001
Breed:Other	-6.671	17.82	-0.374	0.708
Breed:GSD	-172.6	25.79	-6.693	<0.001
Breed:GR	-35.96	15.3	-2.35	0.019
Breed:GRxL	10.32	11.14	0.927	0.354
Breed:LxGR	27.64	16.2	1.706	0.088
Breed:LxGR*	-23.79	25.81	-0.922	0.357

Table 3: Withdrawal reasons and associated numbers for the Old withdrawal group

Withdrawal reason	Number
old	6465

Table 4: Withdrawal reasons and associated numbers for the Attentiveness withdrawal group

Withdrawal reason	Number
attentiveness - low - task focus	47
attentiveness - low - handler focus	29

Table 5: Withdrawal reasons and associated numbers for the Chasing withdrawal group

Withdrawal reason	Number
distraction - high [animals - birds]	128

Table 6: Withdrawal reasons and associated numbers for the Distraction withdrawal group

Withdrawal reason	Number
distraction - high - general	33
distraction - high [objects -food]	22
distraction - high - sounds	4
distraction - high - people	2
distraction - high - scents	2

Table 7: Withdrawal reasons and associated numbers for the Environmental Anxiety withdrawal group

Withdrawal reason	Number
stress resilience - low	178

Withdrawal reason	Number
suspicion - high - sounds	47
suspicion - high - general	43
confidence - low - adaptability	29
suspicion - high - objects	24

Table 8: Withdrawal reasons and associated numbers for the Excitability withdrawal group

Withdrawal reason	Number
social behaviour [hyperactivity - boisterous]	36

Table 9: Withdrawal reasons and associated numbers for the Fear/Aggression withdrawal group

Withdrawal reason	Number
aggression - people	114
aggression - animals	103
suspicion - high - people	6
suspicion - high - animals	3

Table 10: Withdrawal reasons and associated numbers for the Social Behaviour withdrawal group

Withdrawal reason	Number
social behaviour [spending - spends indoor]	50
other - unacceptable post qualification habits	48
social behaviour [scavenge - scrounger]	24

Withdrawal reason	Number
social behaviour - destructive	12
social behaviour ? coprophagia	6
social behaviour - noisy when left	4

Table 11: Withdrawal reasons and associated numbers for the Willingness/Confidence withdrawal group

Withdrawal reason	Number
willingness - low	290
confidence - low decision making	21
